# Qualitative properties and relations

**DOI:** 10.1007/s11098-021-01708-y

**Published:** 2021-08-10

**Authors:** Jan Plate

**Affiliations:** Università della Svizzera italiana, Via Giuseppe Buffi 13, 6900 Lugano, Switzerland

**Keywords:** Properties, Relations, Qualitativeness, Metaphysical perspicuity, Qualitative indiscernibility

## Abstract

This paper is concerned with two concepts of qualitativeness that apply to intensional entities (i.e., properties, relations, and states of affairs). I propose an account of *pure* qualitativeness that largely follows the traditional understanding established by Carnap, and try to shed light on its ontological presuppositions. On this account, an intensional entity is purely qualitative iff it does not ‘involve’ any particular (i.e., anything that is not an intensional entity). An alternative notion of qualitativeness—which I propose to refer to as a concept of *strict* qualitativeness—has recently been introduced by Chad Carmichael. However, Carmichael’s definition presupposes a highly fine-grained conception of properties and relations. To eliminate this presupposition, I tentatively suggest a different definition that rests on a concept of *perspicuous denotation*. In the penultimate section, both concepts of qualitativeness are put to work in distinguishing between different ‘grades’ of qualitative discriminability.

## Introduction

One of the central concepts of David Lewis’s metaphysics was that of *duplicate*: he employed it, e.g., in his explication of the two doctrines of ‘Determinism’ and ‘Materialism’ (i.e., physicalism) in his ‘New Work for a Theory of Universals’ (1983). Thus he proposed that *Determinism* be understood as the thesis that no two possible worlds ‘conform perfectly to the laws’ of our world while (i) failing to be duplicates and yet (ii) having ‘duplicate initial temporal segments’ (pp. 359f.). And he proposed that *Materialism* be understood as the thesis that, ‘[a]mong worlds where no natural properties alien to our world are instantiated’, any two worlds that ‘are exactly alike physically are duplicates’ (p. 364).

As a first stab at analyzing the concept of duplicate, Lewis suggested that we take two things to be duplicates ‘iff they have precisely the same intrinsic properties’ (p. 355); but he noted that we are then faced with the difficult problem of explicating the intrinsic/extrinsic distinction. His preferred alternative—what he took to be the ‘proper course’—was to appeal instead to the notion of *naturalness*, and to say that ‘[t]wo things are qualitative duplicates if they have exactly the same perfectly natural properties’ (p. 356). Later he proposed a more sophisticated account, according to which two things are duplicates just in case ‘(1) they have exactly the same perfectly natural properties, and (2) their parts can be put into correspondence in such a way that corresponding parts have exactly the same perfectly natural properties, and stand in the same perfectly natural relations’ (1986: 61). Whether or not one accepts this latter account (one might for instance wonder how it can apply to abstract objects^1^), Lewis’s first stab—that two things are duplicates iff they have precisely the same intrinsic properties—still seems worth taking seriously. But it also faces a significant shortcoming. For at least under a suitably generous ontology of properties, there are intrinsic properties, such as *being Joe Biden*, that no two things could share. For example, given that *being Joe Biden* is intrinsic, nothing could be a duplicate of Biden in a sense that requires the sharing of all intrinsic properties. This point of course generalizes to all other entities as long as, for any entity *x*, there exists the intrinsic but unshareable property of *being identical with* *x*. In this way the right-hand side of Lewis’s original analysis turns out to be too demanding.

Fortunately, a natural remedy suggests itself: one could appeal to something like Carnap’s (1947) concept of a *purely qualitative* property, and say that two things are duplicates iff they have exactly the same *purely qualitative* intrinsic properties.^2^ Assuming (as most would agree) that the property of *being Biden* is not purely qualitative, a duplicate of Biden, in this revised sense of ‘duplicate’, will no longer be required to share with him that unshareable property. So here we have *one* application of the concept of a purely qualitative property. In the rest of this paper I will use the term ‘pure’ as shorthand for ‘purely qualitative’, while the term ‘impure’ will be used as a synonym of ‘not purely qualitative’.^3^

The distinction between the purely qualitative and the impure may plausibly be applied not only to properties, but also to relations. We may thus speak of purely qualitative and impure *attributes*, where an ‘attribute’ is anything that is either a property or a relation.^4^ Indeed the distinction may be naturally applied also to states of affairs; but, to simplify the exposition, I shall for the most part focus on attributes.

The notion of a purely qualitative attribute is useful in explicating several concepts of discriminability, roughly on the model of Quine’s (1976) threefold distinction between strong, moderate, and weak discriminability. Quine explicated the mentioned concepts in terms of patterns of satisfaction of open sentences of a ‘given interpreted formal language’ (p. 113). Thus he proposed that two objects be called ‘strongly discriminable’ iff in that language ‘there is an open sentence, in one free variable, that is satisfied by one of the objects and not the other’ (*ibid.*). He further proposed to call two objects ‘moderately discriminable’ iff ‘there is an open sentence in two free variables that is satisfied by the two objects in one order and not in the other order’ (*ibid.*). And finally, he proposed that two objects be called ‘weakly discriminable’ iff there exists an open sentence with *two* free variables that is satisfied ‘by the two objects but not by one of them by itself’ (p. 115). We can do something similar—without having to relativize to languages—by talking instead about pure attributes. I will briefly elaborate on this in Sect. 4.^5^

Given its usefulness, it is certainly desirable to have a clear understanding of the distinction between the purely qualitative and the impure. Indeed the extant literature, starting with Carnap, contains a fair number of proposed analyses or definitions. Perhaps the most elegant of these has been put forward by Edward Khamara (1988: 145). The analysis to be formulated in Sect. 2 below will be very similar to Khamara’s proposal: An attribute is purely qualitative just in case it does not ‘involve’ any particular, where the relevant notion of involvement is spelled out in terms of relational instantiation. (Meanwhile a *particular* will be understood to be anything that is neither an attribute nor a state of affairs, or in other words: anything that is not an *intensional entity*.)

While the notion of pure qualitativeness is useful enough, it may reasonably be wondered whether the importance it assigns to the concept of a particular does not constitute something of a liability. For let *A* be any pure attribute, and let *s* be any pure state of affairs. Then, at least *prima facie*, the properties of *being identical with* *A* and *being identical with* *s* will again be pure (provided, as is plausible, that the identity relation does not involve any particular). Moreover, these properties are plausibly regarded as intrinsic. So, on the one hand, any two purely qualitative intensional entities appear to *fail* to be duplicates, at least if we explicate the concept of duplicate in the way suggested above. After all, each member of such a pair has a pure intrinsic property that the other member lacks. But, on the other hand, it may be desirable to have a concept of duplicate that does *not* fail to be satisfied by every given pair of numerically distinct pure intensional entities. For instance, a theorist may find it useful to postulate a multiplicity of pure yet (in some sense) pairwise indiscriminable properties in order to make sense of such ‘quantitative’ properties as *having a mass of ten pounds*.^6^ Or she may think that, for whatever reason, *there are no particulars* and that all physical objects are in fact pure intensional entities.^7^ Let us refer to such a philosopher as a ‘hyper-platonist’. It is not absurd to think that a hyper-platonist may still have need for a non-trivial concept of duplicate, and specifically for a concept under which it is not the case that no two things are duplicates. Recall, for instance, the use to which Lewis has put the concept of duplicate in clarifying the two theses of Determinism and Materialism. A hyper-platonist who (however implausibly) happens to be also a Lewisian modal realist may well be tempted to take those formulations on board. But she will then find that Determinism is vacuously true; for if no two things are duplicates, then *a fortiori* no two possible worlds have duplicate initial temporal segments. And she will find that Materialism is *false* as long as, among those worlds ‘where no natural properties alien to our world are instantiated’, at least two are physically exactly alike. Neither consequence seems appealing.^8^

For reasons such as these, it seems worthwhile to look for a different sense of ‘qualitative’, under which *being identical with* *x* counts as non-qualitative even if *x* is a purely qualitative intensional entity. Such a concept has recently been introduced by Chad Carmichael (2016: §1), who explicates it in the context of a highly fine-grained conception of intensional entities, of the kind developed by Bealer (1982; 1993; 1998), Zalta (1983; 1988), and Menzel (1986; 1993). In Sect. 3, I propose to refer to this concept as one of *strict* qualitativeness and address the question of whether it can be made sense of also on the background of a (moderately) *coarse*-grained conception of intensional entities. I tentatively propose that it can, using a concept of *perspicuous denotation*. However, the task of explicating this latter concept will be left for another paper.

## Purely qualitative

According to Carnap (1947: 138), the purely qualitative properties are those that ‘can be expressed without the use of individual constants, but not without primitive predicates’. The ‘primitive predicates’ are here supposed to be ‘logically independent of each other’ and may be thought of ‘as designating directly observable qualities or relations’.^9^ In contrast to Carnap’s talk of properties, Hempel & Oppenheim (1948) apply the term ‘purely qualitative’ instead to *predicates*, counting a predicate as ‘purely universal, or, as we shall say, purely qualitative, in character’ just in case ‘a statement of its meaning does not require reference to any one particular object or spatio-temporal location’ (p. 156). In these two proposed definitions, we can identify two common themes. The first and possibly most salient is what one might call a ‘linguistic approach’, as both Carnap, on the one hand, and Hempel and Oppenheim, on the other, make explicit use of linguistic notions. The second theme is a reference to the concrete, or ‘the particular’. This is more obvious in the case of Hempel and Oppenheim, who explicitly speak of reference to a ‘particular object or spatio-temporal location’. It is relatively implicit in Carnap’s case, who instead only talks of ‘individual constants’. However, in the formal language that Carnap operates with, individual constants are supposed to designate ‘individuals’, which in turn are ‘best regarded [...] as positions (like space-time points in our actual world)’ (p. 134).

Authors working in the 1970s and later, such as Loux (1974; 1978), Fine (1977), Adams (1979), and Khamara (1988), have tended to pursue *non*-linguistic approaches;^10^ but a ‘reference to the particular’ can be found also in their analyses. Thus Loux (1974: 774f.) takes a property (a ‘universal’) to be impure just in case it ‘incorporates at least one determinate object’. Fine informally characterizes purely qualitative properties as those whose ‘identity does not depend upon the identity of any particular individuals’.^11^ Adams, after first providing an informal characterization in linguistic terms (*op. cit.*, p. 7), proposes an account on which a property is purely qualitative—a *suchness*—iff it is either a ‘basic suchness’ or constructed, by various logical and other operations, from basic suchnesses. For a property to be a *basic suchness* is, in turn, to satisfy three conditions, the second of which is to the effect that the property in question should not be one ‘of being related in one way or another to one or more particular individuals (or to their thisnesses)’.^12^ Finally, under Khamara’s definition of ‘impure’, a property *P* is impure iff ‘there is at least one individual, *y*, such that, for any individual, *x*, *x*’s having *P* consists in *x*’s having a certain relation to *y*’.^13^

### An account

Taken together, these various definitions and analyses suggest that the concept of a purely qualitative (or ‘pure’) attribute may be adequately analyzed along the following lines:

(PQ) An attribute *A* is *purely qualitative* iff it does not involve any particular.

Analogously it may be said that a *state of affairs* is purely qualitative just in case it fails to involve any particular.^14^ For the purposes of this account, the terms ‘attribute’ and ‘particular’ may be formally introduced as follows:

- Something is an *attribute* iff it has an instantiation by one or more, not necessarily pairwise distinct entities (in a particular order). (N.B.: To say that something *has* an instantiation only means that an instantiation of it exists, not that such an instantiation obtains. To have an instantiation is not the same as being instantiated.)
- Something is a *particular* iff it is neither an attribute nor a state of affairs.^15^

The notions of *state of affairs* and *instantiation* are here taken as primitive.

There is now still the question of what exactly it should mean for an attribute or state of affairs to ‘involve’ a given entity. Before we address this, it will help to define some additional terms:

- Something is an *adicity* of an attribute *A* iff it is an ordinal $\alpha >0$ such that *A* has an instantiation by some entities $x_1,x_2,\ldots$ (in this order), with $x_1,x_2,\ldots$ forming a sequence of length $\alpha$.
- A *property* is any monadic (i.e., 1-adic) attribute.
- A *relation* is any $\alpha$-adic attribute for some $\alpha >1$.

A natural way to analyze the notion of involvement in the case of *properties* would be to say that a property *P* involves an entity *x* iff, for some dyadic relation *R*, *P* is the property of *being R-related to* *x*. This would clearly be in line both with Khamara’s definition of ‘impure’ and with the second condition—also mentioned above—of Adams’s definition of ‘basic suchness’.^16^ The generalization to higher adicities, though difficult to express in English, poses no special problem if we make use of $\lambda$-expressions as names for relations. Thus we may say that an $\alpha$-adic attribute *A* (for any ordinal $\alpha >0$) *involves* an entity *y* iff there exists an $(\alpha +1)$-adic relation *R* such that $A=\lambda x_1,x_2,\ldots \,R(x_1,x_2,\ldots ,y)$.^17^ Analogously, we may say that a state of affairs *s* involves an entity *y* iff, for some property *P*, *s* is identical with *P*(*y*), i.e., with *P*’s instantiation by *y*. In other words:

(Inv) An intensional entity (i.e., an attribute or state of affairs) *x* *involves* an entity *y* iff the following two conditions are satisfied:
  (i) If *x* is a state of affairs, then there exists a property *P* such that $x=P(y)$.
  (ii) For any ordinal $\alpha >0$: if *x* is an $\alpha$-adic attribute, then there exists an $(\alpha +1)$-adic relation *R* such that $x=\lambda x_1,x_2,\ldots \,R(x_1,x_2,\ldots ,y)$.

### Attributes and the semantics of $\lambda$-expressions

The main dialectical burden of the above account arguably lies in its requirements on the ontology of relations. For example, if there exists such a thing as the property of *being Biden* (in symbols: $\lambda x\,(x=\mathrm {Biden})$), then there should also exist a relation of identity, lest this property be misclassified as not involving Biden. Similarly, to borrow an example from Khamara (1988: 145n.): if there exists such a thing as the property of *being at an equal distance from Sydney and Melbourne* (which intuitively involves both Sydney and Melbourne), then there should also exist a dyadic relation $R_1$ such that this property is identical with $\lambda x,y\,R_1(x,\text {Sydney})$, as well as a dyadic relation $R_2$ such that the property is identical with $\lambda x,y\,R_2(x,\text {Melbourne})$.

I will here not try to defend the view that attributes are indeed as abundant as the above account requires. However, to make clear just what the account *says*, it will be necessary to provide a semantics of $\lambda$-expressions, together with an at least rudimentary ontology of attributes. (Since this will get fairly technical, it may be advisable, on a first reading, to skip ahead to Sect. 2.3.) To introduce the main ideas, I will be using a toy language $\mathcal {L}$ and develop its semantics and the underlying ontology only as far as necessary to deal with Khamara’s example. In Sect. 2.2.2, I will then briefly consider some problems that arise in connection with adopting a more powerful language and a more expansive ontology.

#### Basic principles

Consider a language $\mathcal {L}$ whose well-formed expressions (or ‘terms’) are constants, variables, and finitely long formulas and $\lambda$-expressions, as follows:

- **Constants:** ‘$$\mathrm {Eq}$$’, ‘$$\mathrm {Mel}$$’, ‘$$\mathrm {Syd}$$’.
- **Variables:** ‘*R*’, ‘*x*’, ‘*y*’.^18^
- **Formulas:** $\ulcorner c(t_1,\ldots ,t_n)\urcorner$, where *c* is a constant or variable and $t_1,\ldots ,t_n$ (with $n>0$) are terms.
- $\pmb {\lambda }$**-Expressions:** $\ulcorner \lambda v_1,\ldots ,v_n\,\varphi \urcorner$, where $v_1,\ldots ,v_n$ (with $n>0$) are pairwise distinct variables and $\varphi$ is a formula such that, for each $i\in \{1,\ldots ,n\}$: $v_i$ has in $\varphi$ at least one free occurrence, but *no* free occurrence at predicate-position.^19^

The requirement that formulas (and hence also $\lambda$-expressions) should be only finitely long is intended to prevent ‘infinite nesting’: each branch of each term’s parse-tree is of only finite length.

Under a certain (at least *prima facie* defensible) ontology of intensional entities, there exists a triadic relation of *being equidistant from *$\ldots$ *and* $\ldots$, whose instantiation by any entities *x*, *y*, and *z*, in this order, is the state of affairs that *x* is at an equal distance from *y* and *z*. Let us call this relation, ‘*E*’, and let us suppose that, for any entities *x*, *y*, and *z*, there does indeed exist an instantiation of *E* by *x*, *y*, and *z*, in this order. We moreover assume that any attribute has *at most one* instantiation by any given sequence of entities.^20^

The simple ontology I have just described may be naturally combined with an almost equally simple semantics of formulas of $\mathcal {L}$. For the sake of brevity, I will write ‘denotes$_g$’ instead of ‘denotes relative to *g*’ (where ‘*g*’ stands for a variable-assignment) and suppress relativization to interpretations of constants. The semantics can then be specified as follows:

1. Relative to any variable-assignment, the constant ‘$\mathrm {Eq}$’ denotes *E*, while the constants ‘$\mathrm {Mel}$’ and ‘$\mathrm {Syd}$’ respectively denote Melbourne and Sydney.
2. For any variable-assignment *g*, variable *v*, and entity *x*: *v* denotes$_g$ *x* iff *g* maps *v* to *x*.
3. For any variable-assignment *g*, constant or variable *c*, entity *x*, positive integer *n*, *n*-adic attribute *A*, terms $t_1,\ldots ,t_n$, and entities $x_1,\ldots ,x_n$: $\ulcorner c(t_1,\ldots ,t_n)\urcorner$ denotes$_g$ *x* iff the following three conditions are satisfied:
  (i) *c* denotes$$_g$$ *A*.
  (ii) For each $i\in \{1,\ldots ,n\}$, $t_i$ denotes$_g$ $x_i$.
  (iii) *x* is an instantiation of *A* by $x_1,\ldots ,x_n$ (in this order).

With this in place, we can proceed to adopt the following assumption about the existence (and uniqueness) of attributes:

(EA) For any variable-assignment *g*, formula $\varphi$, positive integer *n*, and pairwise distinct variables $v_1,\ldots ,v_n$: if the following two conditions are satisfied—
  (i) For each $i\in \{1,\ldots ,n\}$: $v_i$ has in $\varphi$ at least one free occurrence, but *no* free occurrence at predicate-position.
  (ii) $\varphi$ denotes$_g$ a state of affairs.
  — then there exists exactly one attribute *A* such that, for any entity *y*, positive integer *m*, and entities $x_1,\ldots ,x_m$: *y* is an instantiation of *A* by $x_1,\ldots ,x_m$ (in this order) iff $m=n$ and *y* is denoted by $\varphi$ relative to a variable-assignment that is just like *g* except that it maps $v_i$ to $x_i$ for each $i\in \{1,\ldots ,n\}$.

And on this basis we can finally specify the semantics of $\lambda$-expressions:

(S4) For any variable-assignment *g*, formula $\varphi$, entity *x*, positive integer *n*, variables $v_1,\ldots ,v_n$, and $\lambda$-expression *L* with $L=\ulcorner \lambda v_1,\ldots ,v_n\,\varphi \urcorner$: *L* denotes$_g$ *x* iff *x* is an attribute such that, for any entity *y*, positive integer *m*, and entities $x_1,\ldots ,x_m$: *y* is an instantiation of *x* by $x_1,\ldots ,x_m$ (in this order) iff $m=n$ and *y* is denoted by $\varphi$ relative to a variable-assignment that is just like *g* except that it maps $v_i$ to $x_i$ for each $i\in \{1,\ldots ,n\}$.

To see how this works, let *g* be a variable-assignment that maps the variables ‘*x*’ and ‘*y*’ to, respectively, some entities *x* and *y*. Then, by (S1)–(S3) combined with our ontological assumptions, the formula ‘$\mathrm {Eq}(x,y,\mathrm {Mel})$’ denotes$_g$ a state of affairs. By (EA), it then follows that there exists exactly one attribute *A* that is such that, for any entity *y*, integer $m>0$, and entities $x_1,\ldots ,x_m$: *y* is an instantiation of *A* by $x_1,\ldots ,x_m$ (in this order) iff $m=2$ and *y* is denoted by ‘$\mathrm {Eq}(x,y,\mathrm {Mel})$’ relative to some variable-assignment that maps ‘*x*’ to $x_1$ and ‘*y*’ to $x_2$.^21^ By (S4), this attribute is denoted, relative to any variable-assignment, by ‘$\lambda x,y\,\mathrm {Eq}(x,y,\mathrm {Mel})$’. Informally, *A* might be described (albeit awkwardly) as the dyadic relation of *being as far from as from Melbourne*. By similar considerations, the $\lambda$-expression ‘$\lambda x\,\mathrm {Eq}(x,\mathrm {Syd},\mathrm {Mel})$’ also denotes an attribute—and plausibly just the property that we have before referred to as that of *being at an equal distance from Sydney and Melbourne*.

We are now ready to address the question of how the above semantics and ontology help ensure that our account classifies $\lambda x\,\mathrm {Eq}(x,\mathrm {Syd},\mathrm {Mel})$ (a.k.a. *being at an equal distance from Sydney and Melbourne*) as involving both Sydney and Melbourne. In particular, given that $\lambda x\,\mathrm {Eq}(x,\mathrm {Syd},\mathrm {Mel})$ is denoted by ‘$\lambda x\,\mathrm {Eq}(x,\mathrm {Syd},\mathrm {Mel})$’, it follows by (S4) that an entity *y* is an instantiation of $\lambda x\,\mathrm {Eq}(x,\mathrm {Syd},\mathrm {Mel})$ by any given entities $x_1,\ldots ,x_m$ (in this order) iff $m=1$ and *y* is denoted by ‘$\mathrm {Eq}(x,\mathrm {Syd},\mathrm {Mel})$’ relative to a variable-assignment that maps ‘*x*’ to $x_1$. Hence, by (S1)–(S3), an entity *y* is an instantiation of $\lambda x\,\mathrm {Eq}(x,\mathrm {Syd},\mathrm {Mel})$ by any given entities $x_1,\ldots ,x_m$ iff $m=1$ and *y* is the instantiation of *E* by $x_1$, Sydney, and Melbourne, in this order. Crucially, it can now be seen that the same holds for the property $\lambda x\,R(x,\mathrm {Syd})$, where *R* is the dyadic relation $\lambda x,y\,\mathrm {Eq}(x,y,\mathrm {Mel})$ discussed in the previous paragraph. (To save space I delegate the argument to a footnote.^22^) But by (EA) there exists only *one* such attribute. Consequently, the former property, of *being at an equal distance from Sydney and Melbourne*, is nothing other than $\lambda x\,R(x,\mathrm {Syd})$, which means that (Inv) classifies it as involving Sydney. An analogous argument shows that the property is classified as involving Melbourne, as well. This is all as it should be.

#### On expanding the language

The above ontology of intensional entities is clearly quite limited, and so is the formal language that the ontology is built on. It would be natural for a theorist to want to add to $\mathcal {L}$ (as in fact I shall in the following) connectives of negation, conjunction, and disjunction—and, correspondingly, to expand the ontology by admitting ‘negations’, ‘conjunctions’, and ‘disjunctions’ of states of affairs. Certainly one may want to add a device of quantification, and raise the number of available variables. Each such increase in the expressive power of the language, together with the corresponding expansion of the ontology of states of affairs, gives rise, via (EA), to a more expansive view of what attributes there are. But (EA) itself isn’t set in stone. It may for instance be desirable to allow (as in Sect. 2.1 above) for infinitely long argument lists and $\lambda$-prefixes. This would require corresponding changes in (S3), (S4), and (EA). A particularly interesting kind of modification consists in weakening the restrictions that, both in the specification of the syntax of $\lambda$-expressions and in the first numbered clause of (EA), ban variable-occurrences from appearing at predicate-position in the respective formula $\varphi$.

If these latter restrictions were simply lifted altogether, without any compensating changes elsewhere in the system, the resulting version of (EA) would add to our ontology such ‘higher-order’ attributes as the property of *self-instantiation*, which would be denoted by the $\lambda$-expression ‘$\lambda x\,x(x)$’.^23^ This would already be problematic; for on the face of it nothing could decide the question of whether that property instantiated itself. (In particular, we could consistently hold that it does, but could also consistently hold that it doesn’t; and it does not seem as if the matter could be decided by empirical fact.) The situation would be even worse if we also added a negation connective to the language, as this would saddle us with a commitment to a property of *non*-self-instantiation and hence with Russell’s paradox of properties.

In recent ‘higher-order metaphysics’, these difficulties are avoided by means of (some form of) the *simple theory of types* (STT), which takes all things to be organized in a branching hierarchy of non-overlapping types.^24^ On one version of this approach, there is a type *e* of ‘individuals’, a type ${\langle \rangle }$ of ‘propositions’ (or what has here been called ‘states of affairs’), and for any types $\tau _1,\ldots ,\tau _n$, there is a type $\langle \tau _1,\ldots ,\tau _n\rangle$ of *n*-adic attributes that have instantiations by all and only sequences of entities $x_1,\ldots ,x_n$ of types $\tau _1,\ldots ,\tau _n$, respectively. As a result, given that the types don’t overlap, no property has an instantiation by itself. This effectively blocks both of the problems mentioned in the previous paragraph, since each of them requires that *some* property have an instantiation by itself. It also, however, constitutes a rather drastic way of avoiding those problems, and leaves several others—notably, intensional versions of the Epimenides and the Russell–Myhill paradox—unaddressed.^25^ For these reasons it seems advisable to adopt an alternative to STT.^26^ But fortunately, for the purposes of this paper it will not be necessary to weaken the mentioned restrictions, and consequently there is here no need to think very hard about how exactly such a weakening had best be effected.

### Karmo’s problem and Goodman’s riddle

It might be suspected that the account of pure qualitativeness proposed in Sect. 2.1—in particular, the conjunction of (PQ) and (Inv)—presupposes not only a rich ontology of attributes but also that the individuation of attributes should not be ‘too coarse-grained’. Thus consider the following example, which Khamara borrows from Toomas Karmo:

[S]uppose there is an omniscient being, God, who necessarily knows everything; and take any pure property, such as the intrinsic property of being green. Then, necessarily, x is green if and only if x is known by God to be green [...]. (1988: 146)

If we individuated attributes in a way coarse-grained enough that necessary coextensiveness amounted to identity (so that no two attributes would be necessarily coextensive), then the property of *being green* would, under the assumptions of Karmo’s example, count as identical with that of *being known by God to be green*. But under our account, the latter property *involves God*—at least if, for some dyadic relation *R*, the property $\lambda x\,R(x,\mathrm {God})$ is identical with *being known by God to be green*. So *being green* would involve God, and likewise for any other property. If we further assumed that God is a particular, we would be led to the conclusion that under (PQ) every property is impure, which would clearly be undesirable. Humberstone (1996) has called this *Karmo’s problem*.

In fact we might also call it ‘Goodman’s problem’, since it bears a striking resemblance to Nelson Goodman’s (1955: 79f.) attack on the very notion of pure qualitativeness. The similarity is only slightly obscured by the fact that Goodman, as a nominalist, avoids commitment to properties. His crucial premise is to the effect that the predicates ‘green’ and ‘blue’ can be

explained in terms of “grue” and “bleen” and a temporal term; “green”, for example, applies to emeralds examined before time *t* just in case they are grue, and to other emeralds just in case they are bleen. Thus qualitativeness is an entirely relative matter and does not by itself establish any dichotomy of predicates.

Transposed into a platonist key, Goodman’s crucial premise is the thesis that greenness and blueness have metaphysical analyses in terms of grueness, bleenness, and a certain time *t*. In particular, with regard to greenness it might be claimed that this property is nothing other than that of *being either grue and observed before t* *or bleen and not so observed*. If we add the further assumption that there exists such a relation as $\lambda x,y\,\bigl ((\mathrm {grue}(x)\wedge \text {observed-before}(x,y))\vee (\mathrm {bleen}(x)\wedge \lnot \text {observed-before}(x,y))\bigr )$, then by (Inv) it follows that *being green* involves *t*.

To avoid this sort of problem, we apparently have to assume that the individuation of attributes is sufficiently fine-grained: in particular, fine-grained enough to block the identification of *being green* with *being known by God to be green*, as well as the identification of *being green* with *being either grue and observed before t* *or bleen and not so observed*. It would be a mistake, however, to see in this a dialectical burden specific to Sect. 2.1’s account. For, arguably, any view on which attributes are individuated in such a way that *being green* comes out identical with *being known by God to be green* or with *being either grue and observed before t* *or bleen and not so observed* faces the objection that it thereby identifies a property that is intuitively intrinsic and purely qualitative with one that is intuitively extrinsic and impure.^27^ Relatedly, in order for there to exist such a thing as *being known by God to be green*, there plausibly has to be such a thing as God; and in order for there to exist such a thing as *being either grue and observed before* *t* *or bleen and not so observed*, there has to exist the time *t* (which we can perhaps think of as some kind of event). By contrast, the existence of *being green* seems to require the existence neither of a deity nor of a specific time.^28^ So, apparently, it should be identified with neither of the former two properties. If this is right, then the task of avoiding these identifications is a task (if not a problem) for everyone.

This concludes my discussion of pure qualitativeness. The next section will be concerned with an alternative concept, already foreshadowed at the end of the Introduction. As for the notions of pure qualitativeness and involvement, I will in the following assume that the analyses proposed in Sect. 2.1 are correct.

## Strictly qualitative

On some views, some or all of the things that we ordinarily think of as making up the physical world—ships and cabbages, bathtubs and bowsprits—are in fact *events*.^29^ For example, it might be held that Joe Biden is best thought of as the joint life-constituting activity, beginning some time in 1942, of certain Biden-shaped collections of molecules (different collections at different times). In addition, it might be maintained that events form a special class of states of affairs.^30^ For one might think that any life-constituting activity of any collections of molecules over a given span of time is simply a very long conjunction of facts as to which molecules are located where at what times. A similar story could be told for those molecules themselves, so that they, too, would be conceived of as long conjunctions of facts. But all this would of course not mean that *being Biden* is a purely qualitative property. For it may well be the case that the metaphysical analysis of any given person or (other) material object eventually ‘bottoms out’ in particulars of some sort, be they subatomic particles, spacetime points, strings, monads, God, or whatever.^31^ Our abundant ontology of relations will then do the rest in ensuring that *being Biden* is classified as impure. For example, if Biden himself is a conjunction of facts $t_1\wedge t_2\wedge \cdots$, where $t_1$ is the instantiation of a certain property *P* by a certain molecule *M*, and *M* in turn a conjunction of facts $m_1\wedge m_2\wedge \cdots$, where $m_2$ is the instantiation of a certain property *Q* by a certain particular *a*, then the property of *being Biden* will be nothing else than that of *being R-related to* *a*, where *R* is the relation

$$\begin{aligned} \lambda x,y\,\Bigl (x=\bigl (P(m_1\wedge Q(y)\wedge m_3\wedge m_4\wedge \cdots )\wedge t_2\wedge t_3\wedge \cdots \bigr )\Bigr ). \end{aligned}$$

To be sure, it might also be held that the ontology of persons and (other) physical objects does *not* bottom out in particulars—that the world consists of intensional entities ‘all the way down’, with the fundamental level (if there is one) being itself made up of attributes and/or states of affairs. On such a view, *being Biden* is purely qualitative. There is, however, reason to think that it would be desirable to have a somewhat narrower concept of qualitativeness. For one might wish to classify properties like *loving the color red* or *being identical with the identity relation* as in some sense non-qualitative (or ‘impure’), despite the fact that they seem to involve only intensional entities.^32^ Similarly, one might wish to have a concept of qualitativeness under which it is *not* the case that purely qualitative intensional entities cannot have duplicates in the sense of having all their qualitative intrinsic properties in common. (Cf. the relevant remarks at the end of the Introduction.) Even a theorist who can foresee no real use for such a concept, over and above whatever uses she may have for that of pure qualitativeness, may be interested in knowing whether such a concept is available.

### Carmichael’s definition of ‘qualitative’

A concept of the sort just described has recently been introduced by Carmichael:

The property *being identical to Socrates* is impure because it involves a non-property, Socrates. But the property *being identical to redness* is not impure in this sense—it involves nothing that is not a property. Is it qualitative? I claim it is not. Contrast the way that redness is involved in *being identical to redness* to the way in which it is involved in the property of *being red and round*. In the latter case, but not the former, there is an intuitive sense in which redness occurs *predicatively*. (2016: 311; original italics)

In defining his concept of qualitativeness, Carmichael follows ‘the algebraic approach to properties, relations, and propositions’ developed by Bealer (1982; 1993; 1998), Zalta (1983; 1988), and Menzel (1986; 1993), under which ‘properties and relations are analyzed by appeal to primitive logical operations—negation, conjunction, disjunction, predication, and so on—on a domain of properties, relations, propositions, and individuals’ (p. 312). On this background, he first defines a notion of *non-predicative occurrence* by saying that a property $F_1$ occurs non-predicatively in a property $F_2$ just in case ‘$F_1$ is a constituent of $F_2$, and, in the analysis of $F_2$, $F_1$ does not appear as a subject operand in the application of the predication operation’ (*ibid.*, italics added).^33^ The concept of a *qualitative* property is then defined by saying that a property *F* is qualitative iff ‘no constituent of *F* occurs in *F* non-predicatively’.

In Carmichael’s framework, any ‘application of the predication operation’ has as its ‘subject operand’ some attribute *A* and yields as output an instantiation of *A* by the other operands (in a particular order). Let us call these other operands the *object operands* of the respective application. According to Carmichael, the property of *being identical with the color red* is non-qualitative because there exists at least one entity—namely, the color red (or ‘redness’)—that occurs in it non-predicatively: for while redness is a constituent of that property, it does not, in the latter’s analysis, appear as a subject operand in the application of the predication operation. So far, so good.

Unfortunately, Carmichael’s definition has unintended consequences. For example, consider the property of *being identical with the identity relation*. While this should fall on the *non*-qualitative side of the distinction, Carmichael’s definition classifies it as qualitative. This is because, in the analysis of that property, the identity relation appears as a subject operand of the predication operation, and hence does not (under Carmichael’s definition) occur non-predicatively in that property; and—at least barring metaphysical surprises—no other entity occurs in it non-predicatively, either. Similar remarks apply, e.g., to the property of *being red while loving the color red*.

An obvious way to repair this defect is to revise the definition of ‘occurs non-predicatively’. Rather than to say that a property $F_1$ occurs non-predicatively in a property $F_2$ iff $F_1$ is a constituent of $F_2$ and does not, in the analysis of $F_2$, appear ‘as a subject operand in the application of the predication operation’, we could just say that $F_1$ occurs non-predicatively in $F_2$ iff, in the analysis of the latter, $F_1$ appears as an *object operand* in the application of the predication operation. Under this revised definition of ‘occurs non-predicatively’, and leaving Carmichael’s definition of ‘qualitative’ otherwise unchanged, *being identical with the identity relation* comes out non-qualitative (as does *being red while loving the color red*).^34^

### Structural involvement

On a suitably fine-grained conception of attributes, the definition just proposed may be entirely adequate. It starts to run into trouble, however, once we adopt a somewhat coarse-grained conception—such as one under which every property *P* is identical with that of *having P* *and being such that P* *is self-identical*. (The latter may be symbolized as ‘$\lambda x\,(P(x)\wedge I(P,P))$’, with ‘*I*’ denoting the identity relation.) This identification can be motivated by the thought that, for any given object *x*, to have *P* while being such that *P* is self-identical requires of *x* nothing more and nothing less than what is required of *x* in order for it to have *P*. In other words: once you have *P*, the ‘additional’ requirement of being such that *P* is self-identical becomes entirely trivial. A distinction between *P* and $\lambda x\,(P(x)\wedge I(P,P))$ would be a “distinction without a difference”. (Even though the issue is controversial, I think that the intuitive pull is hard to deny.^35^) But now, under a conception of attributes that is coarse-grained enough to *reject* this distinction, every property will occur non-predicatively in itself (under the revised definition),^36^ so that any property whatsoever will turn out to be non-qualitative. For an adherent of this sort of conception, the present distinction between qualitative and non-qualitative properties will as a result be rather useless. So it may be worth asking whether we can draw the distinction in such a way that it avoids triviality even on the background of a (within reason) coarse-grained conception of attributes. Let me briefly try to indicate a possible way to do this.

Various philosophers have made appeal to a notion of *perspicuity*, either as applied to whole languages or to particular expressions.^37^ Somewhat more concretely, one might think that intensional entities can be meaningfully said to be *perspicuously denoted* by particular expressions of a certain formal language (relative to a given variable-assignment and interpretation of its non-logical vocabulary). For now, let us suppose that the formal language in question is a variant of the language $\mathcal {L}$ described in Sect. 2.2. Then, with the help of a suitable concept of perspicuous denotation, applicable to the terms of this language, a new notion of involvement may be introduced as follows:

(SInv) An intensional entity *x* *structurally involves* an entity *y* iff there exist a term *t* and a variable-assignment *g* that satisfy the following two conditions:
  (i) *t* perspicuously denotes$$_g$$ *x*.
  (ii) *t* contains, as an element of an argument list, a free occurrence of a term that denotes$_g$ *y*.

On this basis, we may next say that an intensional entity is *strictly qualitative* iff it does not structurally involve anything; otherwise it may be said to be *weakly impure*. Let us apply this distinction to a few of the above examples. To begin with, suppose that the property of *being identical with the identity relation* can be perspicuously denoted by ‘$\lambda x\,I(x,I)$’. It then follows from (SInv) that this property structurally involves the identity relation and is hence weakly impure. Analogously for *loving the color red*, only that here the $\lambda$-expression in question would have to be a good deal more complicated—at least if it is correct to assume that, in a $\lambda$-expression that perspicuously denotes *loving the color red*, the *loving* relation and the color red will not be represented by mere constants or variables.

The great question is now how to define (or analyze) the relevant concept of perspicuous denotation. It won’t be possible to address this in the present paper, but the remarks at the beginning of this subsection point to some desiderata that can serve as clues. Recall for instance that, under a certain (not implausibly) coarse-grained conception of attributes, every property *P* is denotable by the $\lambda$-expression ‘$\lambda x\,(P(x)\wedge I(P,P))$’, in which the variable ‘*P*’ occurs as an element of an argument-list. Hence, if we are to avoid the unwelcome result that every property whatsoever structurally involves itself (and thereby fails to be strictly qualitative), then it had better not be the case that every property *P* is *perspicuously* denotable by ‘$\lambda x\,(P(x)\wedge I(P,P))$’.

If the concept of perspicuous denotation can be made precise, it may be tempting to use it also in an account of *pure* qualitativeness. In particular, one might suggest that an intensional entity *x* be called purely qualitative iff it is not the case that there exist some term *t* and variable-assignment *g* such that: (i) *t* perspicuously denotes$_g$ *x*, and (ii) *t* contains a free occurrence of a term that denotes$_g$ a particular.^38^ Suppose moreover that (as is the case with $\mathcal {L}$) the underlying language is such that, for any terms $t_1$ and $t_2$, and any variable-assignment *g*: if $t_1$ has a denotation relative to *g*, and $t_2$ denotes$_g$ a particular, then any free occurrence of $t_2$ in $t_1$ is an element of an argument list. On this supposition it is straightforward to see that any intensional entity that *fails* to be purely qualitative, under the account just described, will structurally involve a particular, and will therefore fail to be strictly qualitative. By contraposition, any intensional entity that is strictly qualitative will then also be *purely* qualitative.

## Grades of qualitative discriminability

According to what has been claimed in the Introduction, the notion of a purely qualitative attribute allows us to introduce different grades of qualitative discriminability, roughly in the manner of Quine, but without having to relativize to languages. The main purpose of this section is to substantiate that claim by providing the respective definitions. A subsidiary motive is to indicate an additional use case for the concept of perspicuous denotation.

If the concept of pure qualitativeness allows us to introduce different grades of qualitative discriminability, it stands to reason that the notion of *strict* qualitativeness discussed in the previous section can be put to exactly the same use. We will thus have two related sets of grades of discriminability, explicated in exactly analogous ways with the help of the concepts of pure and strict qualitativeness, respectively. To save space, I shall write ‘p. q.’ to abbreviate ‘purely qualitative(ly)’ and ‘s. q.’ to abbreviate ‘strictly qualitative(ly)’.

Before we turn to the Quinean notions, let us say that two things are *intrinsically purely (strictly) qualitatively discriminable* iff one of them has a p. q. (s. q.) intrinsic property that the other lacks. Relatedly, two things may be said to be *purely* (*strictly*) *qualitative duplicates* just in case they fail to be intrinsically p. q. (s. q.) discriminable. For example, on the assumption that *being red* is purely qualitative, it will plausibly be the case that *being identical with redness* is also purely qualitative (as well as intrinsic), so that *being red* and *being green* (say) will be intrinsically p. q. discriminable and consequently not be p. q. duplicates. Nonetheless, for all that has been said here, *being red* and *being green* may well fail to be intrinsically *strictly* qualitatively discriminable, and may accordingly turn out to be s. q. duplicates. Given a suitably precise notion of fundamentality, it might be an interesting metaphysical question whether the fundamental properties form an equivalence class under intrinsic s. q. indiscriminability.

Next, hewing fairly closely to Quine’s own definition, we may say that two entities are *strongly purely* (*strictly*) *qualitatively discriminable* iff one of them has a p. q. (s. q.) property that is not had by the other.^39^ Since this definition does not require the properties in question to be intrinsic, the notions thus defined are a good deal weaker than the corresponding concepts of *intrinsic* discriminability.

Reference to qualitative *relations* comes into play as we turn to Quine’s two weakest notions of discriminability. Two entities *x* and *y* may be said to be *moderately purely* (*strictly*) *qualitatively discriminable* iff there exists a p. q. (s. q.) dyadic relation *R* such that it is not the case that: *x* bears *R* to *y* iff *y* bears *R* to *x*. Unfortunately, as Linnebo & Pettigrew (2012: §10) have shown, this definition offers no guarantee that moderate p. q. (s. q.) *in*discriminability, understood as the failure to be moderately p. q. (s. q.) discriminable, is a transitive relation. This is problematic, as ordinarily one would like one’s indiscriminability relations to be *a priori* transitive.

No such worry arises in the case of *weak* discriminability.^40^ To a first approximation, two entities may be said to be weakly purely (strictly) qualitatively discriminable iff there exists a p. q. (s. q.) dyadic relation that at least one of them bears to the other but not to itself. The reason why this is only an approximation has to do with the fact that, on the highly plausible assumption that the relation of numerical distinctness is purely (strictly) qualitative, weak p. q. (s. q.) discriminability will, under the definition just suggested, coincide with plain numerical distinctness. To avoid this outcome, we have to add a clause to the effect that the relation in question should not be *distinctness-entailing*. On the background of a suitably coarse-grained conception of attributes, this latter concept may be defined by saying that a dyadic relation *R* is distinctness-entailing just in case *R* is identical with $\lambda x,y\,(R(x,y)\wedge (x\not =y))$.

However, merely to require that *R* should not be distinctness-entailing is not yet enough.^41^ To see this, let *x* and *y* be any two entities, and let *Q* be some p. q. (s. q.) dyadic relation that *x* bears to itself and whose ‘negation’ $\lambda x,y\,\lnot Q(x,y)$ is not distinctness-entailing.^42^ Then *x* bears to *y* but not to itself the relation $\lambda x,y\,\lnot ((x=y)\wedge Q(x,y))$, which is not distinctness-entailing. Hence, unless we impose some additional requirement, any two entities will come out weakly p. q. (s. q.) discriminable. The question is what this requirement might be.

A natural and (as far as I can see) promising candidate is the constraint that the relation in question should not be *disjunctive*, where ‘disjunctive’ might be defined by making use of the concept of perspicuous denotation, somewhat as follows:^43^

(Dis) An intensional entity *x* is *disjunctive* iff there exist a term *t* and a variable-assignment *g* that satisfy the following two conditions:
  (i) *t* perspicuously denotes$$_g$$ *x*.
  (ii) *t* contains at least one occurrence of ‘$\wedge$’ that (a) stands in the scope of an odd number of occurrences of ‘$\lnot$’ and (b) is *not* contained in a term-occurrence that stands at argument-position.^44^

Assuming that the above relation $\lambda x,y\,\lnot ((x=y)\wedge Q(x,y))$ can be perspicuously denoted by something of the form ‘$\lambda x,y\,\lnot ((x=y)\wedge \cdots )$’, this definition will classify it as disjunctive.

With the help of the concept of disjunctiveness, we may say that two things are *weakly purely* (*strictly*) *qualitatively discriminable* iff there exists a p. q. (s. q.) dyadic relation *R* such that: (i) at least one of those things bears *R* to the other but not to itself, and (ii) *R* is neither distinctness-entailing nor disjunctive. Max Black’s (1952) two iron spheres in an otherwise empty universe form what is probably the best-known example of two (hypothetical) objects that may plausibly be regarded as at least weakly p. q. discriminable—since the relation of spatial distance that is stipulated to hold between them may be plausibly regarded as at least purely (if not also strictly) qualitative—while being neither strongly p. q. nor strongly s. q. discriminable. A slightly less famous example comes from the theory of complex numbers, in the form of *i* and $-i$, the two square roots of minus one.^45^

## Conclusion

The goal of this paper has been not so much to defend a specific set of theses as to obtain a clearer view of a certain part of the conceptual landscape. Historically, considerable attention has been given to the notion of a purely qualitative property (or relation). I have suggested an analysis of this notion that follows in the footsteps of traditional accounts, in particular Khamara’s (1988), and have tried to shed some light on the presuppositions of this analysis with regard to the ontology of intensional entities. I have then tried to draw attention to a different (though related) concept—that of *strict* qualitativeness—which, unlike the concept of *pure* qualitativeness, does not rest on the notion of a particular. While Carmichael’s definition of ‘qualitative’ goes some way towards clarifying this alternative concept, it presupposes a highly fine-grained conception of properties and relations. To dispense with this presupposition, I have suggested an analysis in terms of perspicuous denotation. As we have seen, this latter notion promises to be useful also in defining a concept of disjunctiveness (and hence, indirectly, in defining concepts of weak qualitative discriminability); but more work is needed to make it precise.

## Footnotes

1 Oliver (1996: 43).

2 Cf. Lewis (1986: 62) ‘Two things are duplicates iff they have the same intrinsic qualitative character’. However, rather than to analyze the notion of intrinsic qualitative character in terms of two separate notions of intrinsicality and qualitativeness, Lewis goes on to say that two things’ having the same intrinsic qualitative character is ‘a matter of the perfectly natural [...] properties of those things and their parts, and of the perfectly natural external relations of their parts’. While I shall not be relying on a concept of perfect naturalness in this paper, the notion of *perspicuous denotation* that will become relevant in Sect. 3.2 may not implausibly be elucidated on the basis of a concept of fundamentality that is applicable to properties and relations (and states of affairs): a concept that is at least closely related to that of perfect naturalness.

3 The term ‘qualitative property’ has been used in a variety of senses by different authors. Yablo (1995) applies the adjective ‘qualitative’ to those properties that are ‘secondary and phenomenal’ (p. 478). Bricker (2006), writing within the context of what he calls ‘Leibnizian’ modal realism, identifies the ‘fundamental qualitative’ properties and relations with ‘those that are subject to principles of recombination’ (p. 50), which he contrasts with those that—like identity, parthood, and instantiation—‘are needed to provide the underlying framework for logical space’ (p. 49). (This usage may be inspired by a passage in Lewis [1986: 62f.] that can be read as suggesting that ‘qualitative character’ consists of ‘properties that are, though not perfectly natural, still somewhat natural in virtue of their definability from perfectly natural properties and relations’. Eddon [2011: 320] refers to a similar characterization of the qualitative properties as the ‘standard strategy’; also cf. Cowling [2015: 292f.] and Dorr [2019: §4.3].) Cowling (*op. cit.*) lists four different classes whose members he considers ‘paradigmatic non-qualitative properties’ (p. 283), with only one of these classes consisting of impure properties. Going back somewhat further, one can find that even the term ‘purely qualitative’ has been used in a number of different ways. For instance, Chisholm (1984: 8) understands it as designating a certain ‘logical characteristic’ that, ‘so far as anyone knows, is peculiar to what is psychological or mental’, while Martin (1996: 85) calls a property ‘purely qualitative’ just in case it is ‘without any implications of dispositionality’. But the mainstream usage seems to be largely aligned with Carnap. (Cf. Sect. 2 below.)

4 In Sect. 2.1 I will offer a more formal definition of ‘attribute’ and reverse the order of analysis, defining ‘property’ and ‘relation’ in terms of ‘attribute’.

5 Cowling (2015: §2) and Hoffmann–Kolss (2019: §1) discuss further applications. The notion of a purely qualitative *fact* figures prominently in Dasgupta (2017). The original ‘use case’ for the distinction was Carnap’s (1947: 146) tentative proposal, in response to Goodman (1946), that all and perhaps *only* purely qualitative properties are ‘projectible’, roughly in the sense of allowing for inductive inference. (For discussion of the larger historical context, see, e.g., Schwartz [2011] and references therein.) Under Carnap’s definition, a property is purely qualitative iff it ‘can be expressed without the use of individual constants, but not without primitive predicates’ (p. 138). Here the ‘primitive predicates’ may be imagined ‘as designating directly observable qualities or relations’ (p. 135f.), and Carnap moreover requires that ‘[t]he qualities and relations designated by the primitive predicates must not be analyzable into simpler components’ (p. 136). Goodman, in his reply, held such talk of analyzability to be ‘meaningful only with respect to a sphere of reference and a method of analysis’ (1947: 149). But even apart from Goodman’s qualms about absolute analyzability, the idea that all purely qualitative properties are projectible ought arguably to be rejected, as has been pointed out by Cohen (1989: 198f.). For example, consider the ‘grue-like’ property of *being either green and observed before the first instance of interstellar travel or blue and not so observed*. Surely this property is not projectible; but for all we know it may well be purely qualitative in Carnap’s sense.

6 Cf. Rodriguez-Pereyra (2017).

7 See, e.g., Fitch (1971), Paul (2017), Builes (2021). The Pythagorean view that our universe is at bottom a universe of mathematical entities is of a broadly similar character. A version of this view has recently been defended (on Quinean grounds) by Kemp (2017). The physicist Tegmark (1998; 2008) has defended the related view that the universe is itself a mathematical object. Some other views, such as the one espoused by Dasgupta (2009), also deny the existence of particulars but—rather than to identify physical objects with pure attributes or states of affairs—entail that (at least in a certain ‘fundamental’ sense) there are no physical objects in the first place. For related discussion, see, e.g., Turner (2017), Dasgupta & Turner (2017), Bacon (2019a), and Sider (2020: ch. 3).

8 It is worth keeping in mind here that Materialism is supposed to be a theory of mind; it is *not* supposed to be incompatible with hyper-platonism. One can be a hyper-platonist and still coherently wonder whether the mental reduces, in some sense, to the physical. (Admittedly, neither Lewis’s formulation of Determinism nor his formulation of Materialism is uncontroversial, and both may be best regarded as only approximately adequate. Cf., e.g., Hoefer [2016] on determinism and Stoljar [2021] on physicalism.)

9 *Op. cit.*, p. 135f. It is worth noting that Carnap’s talk of expressibility is to be understood as referring to expressibility in a certain regimented language.

10 An exception is Goldstick (1986), who takes as his target the notion of a ‘purely qualitative term’. An expression (as used in a given context) is said to be purely qualitative just in case it is not ‘definitely referring’ (p. 71). The rest of Goldstick’s paper is largely devoted to the problem of specifying what this means, which leads him to confront some problems of a predominantly linguistic nature.

11 *Op. cit.*, pp. 137 and 174. Fine also provides a more formal characterization, according to which ‘an [intensional] entity is pure iff it stays the same under any automorphism whatsoever’ (*ibid.*). The relevant concept of automorphism is defined on the basis of a modal framework. But in setting up that framework, Fine stipulates that each polyadic predicate of his formal language *L* be ‘purely qualitative’ in the sense that ‘it does not involve a ‘reference’ to any specific individual’ (p. 147). Recently, Andrew Bacon (2019b: 1038) has offered a model-theoretic characterization of qualitativeness that is quite similar to Fine’s but still more abstract: instead of postulating a space of possible worlds, it is purely algebraic.

12 *Op. cit.*, pp. 7f. The other two conditions are as follows: ‘(1) It [the property in question] is not a thisness and is not equivalent to one’ and ‘(3) [It] is not a property of being identical with or related [...] to an extensionally defined set that has an individual among its members, or among its members’ members, or among its members’ members’ members, etc.’. Adams’s proposal bears some structural similarity to an account proposed by Rosenkrantz (1979). A property is impure, according to Rosenkrantz, iff it is either ‘basic impure’ or can be ‘generated out of’ basic impure properties according to ‘an appropriate set of recursive rules’ (p. 523); and a property is basic impure iff it ‘possibly has a concrete constituent’ (p. 520). (Hoffmann-Kolss [2019] has recently put forward a similar account with explicit reference to Rosenkrantz.) A crucial difference to Adams’s proposal lies in the fact that in the latter’s case it is the *pure* properties that are recursively generated out of ‘basic suchnesses’. If one had to opt for an explicitly recursive analysis, Adams’s approach would arguably be preferable: one obtains the non-basic pure properties by combining other pure properties (though Adams, like Rosenkrantz, neglects relations), whereas *im*pure properties may be constructed from a mix of both pure and impure properties (possibly along with other things). This would straightforwardly allow, for instance, that the property of *being either green or identical with Biden* is classified as impure, as intuitively it should be.

13 *Op. cit.*, p. 145 (with slight changes of notation). Khamara’s proposal is superficially quite similar to the account offered by Loux (1978: 133), according to which a property *P* is impure just in case ‘there is some relation, *R*, and some substance, *s*, such that necessarily, for any object, *x*, *x* exemplifies *P* if and only if *x* enters into *R* with *s*’. As Khamara points out, however, this account runs into the difficulty that Humberstone (1996) would later refer to as ‘Karmo’s problem’. (See Khamara *op. cit.*, p. 146. This will be further discussed in Sect. 2.3 below.)

14 Cf. the distinction between ‘singular’ and ‘general’ propositions in, e.g., Kaplan (1975: 724): ‘Let us adopt the terminology *singular proposition* for those (purported) propositions which contain individuals as immediate constituents, and *general proposition* for the others’. The first use (to my knowledge) of the verb ‘to involve’ in an analysis of pure qualitativeness or related notions is due to Fine (1977: 147). (Cf. footnote 11 above.) However, Adams’s (1981: 3) use of the verb comes closer to present usage when he says that ‘all possibilities are purely qualitative except insofar as they involve individuals that actually exist’. Fine himself would use the verb in this way when characterizing a ‘purely qualitative individual essence’ as ‘one not involving any individuals at all, whether possible or actual’ in his (1985: 148n.). In a similar vein, Swoyer (1984: 611) notes that ‘[i]ntuitively, a purely qualitative property is one that does [not] involve any individuals’.

15 Thus states of affairs are non-particulars. This contrasts with the usage employed by, e.g., David Armstrong (1978: 115; 1997: §8.4), who counts states of affairs as ‘particulars’ due to their being ‘unrepeatable’. However, at least in the present context it seems appropriate to lump states of affairs together with attributes—and not only because the notion of pure qualitativeness is applicable to states of affairs just as it is to attributes. Consider, e.g., the property of *being such that *$\xi$ *obtains*, where $\xi$ is the (let us suppose) purely qualitative state of affairs that snow is white. Intuitively, it seems that this property should itself be classified as purely qualitative, and hence as *not* involving a particular; after all, on a not-implausibly coarse-grained conception of attributes, it is identical with *being such that snow is white*, which certainly *looks* purely qualitative. But if $\xi$ were classified as a particular—and if there is such a thing as the relation $\lambda x,y\,(x \text { is such that }y\text { obtains})$, which for the sake of the argument may be assumed to be the case—then our account would end up classifying it as impure.

16 Recall that, according to Khamara, a property *P* is impure iff ‘there is at least one individual, *y*, such that, for any individual, *x*, *x*’s having *P* consists in *x*’s having a certain relation to *y*’. The problem of how the ‘consists in’ locution should be understood has been discussed at some length by Humberstone (1996: §2), who pessimistically concludes that the notion of an impure property (and similarly that of a ‘relational’ property) cannot be successfully analyzed unless one either takes properties to be outright *identical* with concepts or understands any claims about a property’s being pure or impure as relativized to a description (p. 226). He seems to be led to this conclusion in part by the idea that Khamara’s ‘consists in’ locution must be understood as expressing an *asymmetric* concept, in the sense that ‘*A* consists in *B*’ does not in general describe the same situation as ‘*B* consists in *A*’. Although this is certainly correct as far as the locution’s use in English is concerned, one may doubt whether the asymmetry in question plays any essential role in Khamara’s account. For instance, it would seem to do no harm to the account if the ‘consists in’ were replaced with an expression of a suitable symmetric notion, such as identity. (Cf. also Dorr [2016: 80–82].)

17 Essentially the same approach has been taken by Dorr (2016: 80) in his definition of ‘weakly prior’. (As will be clear from the context, ‘$x_1,x_2,\ldots ,y$’ should here be read as abbreviating a list of variable-occurrences of length $\alpha +1$ that has an occurrence of ‘*y*’ as its last element.) The suggested use of $\lambda$-notation might provoke an immediate objection, concerning ‘multigrade’ attributes (i.e., attributes with more than one adicity). Since a $\lambda$-expression typically comes with a fixed number of $\lambda$-variables—meaning the variables listed after the initial ‘$\lambda$’—and hence comes, if read as a predicate, with a fixed number of argument-positions, $\lambda$-expressions do not easily lend themselves to denoting multigrade attributes; and, as a result, the suggested approach is not readily applicable to such attributes. While this does constitute something of a weakness, I think that it can be excused as a (relatively) harmless simplification. (For some recent related discussion, see Dixon [2019: 66].)

18 The distinction between upper- and lower-case variables carries no significance.

19 The notion of a free variable-occurrence should here be understood in the usual way. Similarly for the notion of a term-occurrence’s standing at predicate-position.

20 That is, any attribute *A* has at most one instantiation by any given entity $x_1$, at most one instantiation by any given (not necessarily distinct) entities $x_1$ and $x_2$ (in this order), and so on. (The ‘and’ between ‘$x_1$’ and ‘$x_2$’ is intended to function not as a term-forming operator but as a delimiter.)

21 More carefully, relative to a variable-assignment that $is\, just\, like\, g$ except that it maps ‘*x*’ to $x_1$ and ‘*y*’ to $x_2$. But since ‘*x*’ and ‘*y*’ are the only variables that occur free in ‘$\mathrm {Eq}(x,y,\mathrm {Mel})$’, it does not matter—as can be seen from (S1)–(S3)—what entities *g* assigns to any other variables.

22 By semantic ascent, the property in question is denoted by ‘$\lambda x\,R(x,\mathrm {Syd})$’ relative to a variable-assignment that maps ‘*R*’ to $\lambda x,y\,\mathrm {Eq}(x,y,\mathrm {Mel})$. Let *g* be such a variable-assignment. Then, by (S4) we have that:

1. For any entity *y*, integer $m>0$, and entities $x_1,\ldots ,x_m$: *y* is an instantiation of $\lambda x\,R(x,\mathrm {Syd})$ by $x_1,\ldots ,x_m$ (in this order) iff $m=1$ and *y* is denoted by ‘$R(x,\mathrm {Syd})$’ relative to some variable-assignment that is just like *g* except that it maps ‘*x*’ to $x_1$.

Let now $x_1$ be any entity, and let $g'$ be a variable-assignment that is just like *g* except that it maps ‘*x*’ to $x_1$. Then, by (S2), ‘*R*’ and ‘*x*’ respectively denote$_{g'}$ $\lambda x,y\,\mathrm {Eq}(x,y,\mathrm {Mel})$ and $x_1$. So, by (S1) and (S3), it follows that

(2) An entity *y* is denoted$_{g'}$ by ‘$R(x,\mathrm {Syd})$’ iff *y* is the instantiation of $\lambda x,y\,\mathrm {Eq}(x,y,\mathrm {Mel})$ by $x_1$ and Sydney, in this order.

But by what has been said in the previous paragraph (in the main text), an entity *y* is an instantiation of $\lambda x,y\,\mathrm {Eq}(x,y,\mathrm {Mel})$ by $x_1$ and Sydney, in this order, iff *y* is denoted by ‘$\mathrm {Eq}(x,y,\mathrm {Mel})$’ relative to some variable-assignment that maps ‘*x*’ to $x_1$ and ‘*y*’ to Sydney. And by (S1)–(S3), this is the case exactly if *y* is the instantiation of *E* by $x_1$, Sydney, and Melbourne, in this order. So we have that

(3) An entity *y* is an instantiation of $\lambda x,y\,\mathrm {Eq}(x,y,\mathrm {Mel})$ by $x_1$ and Sydney, in this order, iff *y* is the instantiation of *E* by $x_1$, Sydney, and Melbourne, in this order.

Putting (1)–(3) together (and seeing that $x_1$ was arbitrary), we can infer that, for any $m>0$ and any $x_1,\ldots ,x_m$, an entity *y* is an instantiation of $\lambda x\,R(x,\mathrm {Syd})$ by $x_1,\ldots ,x_m$ (in this order) iff $m=1$ and *y* is the instantiation of *E* by $x_1$, Sydney, and Melbourne, in this order. This is what needed to be shown.

23 To see this, recall that the above ontology admits (among other things) a property $\lambda x\,\mathrm {Eq}(x,\mathrm {Syd},\mathrm {Mel})$. Under our ontological assumptions, this property has an instantiation by itself, which is, relative to a suitable variable-assignment, denoted by the formula ‘*x*(*x*)’. By the modified (EA) together with (S2)–(S3), it then follows that there exists an attribute *A* such that, for any entities $x_1,\ldots ,x_m$ and *y*: *y* is an instantiation of *A* by $x_1,\ldots ,x_m$ iff $m=1$ and *y* is an instantiation of $x_1$ by itself.

24 In the context of STT, the inhabitants of ‘higher’ types are not ordinarily referred to as ‘things’. However, we have to use *some* term, and ‘thing’ seems to work just as well (or as badly) as ‘entity’ or ‘item’. The main contributors to contemporary higher-order metaphysics include Williamson (2013), Dorr (2016), Goodman (2017), and Bacon (2019b; 2020). All of them rely on STT.

25 See, e.g., Thomason (1989) and Church (1993). One could also easily construct an intensional version of Yablo’s (1993) paradox as well as ‘truth-teller’ variants of the paradoxes just mentioned. (See Plate [MSa: §2] for related discussion.) As Uzquiano (2015: 334) has pointed out, Russell’s original version of the Russell–Myhill paradox relies on a premise that is not particularly compelling. But there are other versions of the paradox that do not rest on that premise. For example, on the background of STT (combined with the resources of classical conjunction and negation, quantification over type ${\langle \langle \rangle \rangle }$, identity at types ${\langle \rangle }$ and ${\langle \langle \rangle \rangle }$, and $\lambda$-abstraction over type ${\langle \rangle }$), considerations analogous to those of Russell–Myhill lead to the *prima facie* implausible conclusion that there exists *no* ‘property of properties of states of affairs’ (i.e., no entity of type ${\langle \langle \langle \rangle \rangle \rangle }$) such that, for each entity of type ${\langle \langle \rangle \rangle }$, the property’s instantiation by that entity is distinct from its instantiation by every *other* entity of that type. For suppose that *P* is such a property—i.e., such that, for any entities *x* and *y* of type ${\langle \langle \rangle \rangle }$: if $P(x)=P(y)$, then $x=y$—and let *M* be a property of states of affairs (i.e., an entity of type ${\langle \langle \rangle \rangle }$) such that, for any given state of affairs *x*: *M*(*x*) obtains just in case, for some entity *y* of type ${\langle \langle \rangle \rangle }$, it is both the case that *y*(*x*) fails to obtain and that $x=P(y)$. (In symbols, such a property may be represented as $\lambda x^{\langle \rangle }\,\exists y^{\langle \langle \rangle \rangle }\,\bigl (\lnot y^{\langle \langle \rangle \rangle }(x^{\langle \rangle })\wedge (x^{\langle \rangle }=P(y^{\langle \langle \rangle \rangle }))\bigr )$.) If we then ask whether *P*(*M*) has *M*, we are led to a contradiction. (To see this, suppose first that *P*(*M*) has *M*. Then there is some *y* of type ${\langle \langle \rangle \rangle }$ such that: *y*(*P*(*M*)) fails to obtain and $P(M)=P(y)$. By hypothesis, since $P(M)=P(y)$, it follows that $y=M$. So, since *y*(*P*(*M*)) fails to obtain, *P*(*M*) does not have *M*. This contradicts the initial supposition that *P*(*M*) has *M*; and so we conclude that, indeed, *P*(*M*) does not have *M*. But then, by the way *M* was introduced, *M*(*P*(*M*)) obtains. So *P*(*M*) has *M*, after all, which contradicts the earlier result.)

26 I suggest such an alternative in my (MSa; MSb). On this proposal, the restrictions in question are weakened as follows: Rather than to say that each $v_i$ has (in the respective formula $\varphi$) *no* free occurrence at predicate-position, we only require that, *if* $v_i$ has in $\varphi$ a free occurrence at predicate-position (or at sentence-position, for that matter), then it is *typed*. In a nutshell, a typed variable is one that can only denote intensional entities of an order less than a certain ordinal $\alpha$, which is indicated by a superscript. (The above statement (S2) would need to be modified accordingly.) For example, the variable ‘$x^2$’ can only denote zeroth- or first-order intensional entities. On the resulting ontology (once negation is added into the mix), there does exist a property $\lambda x^1\,\lnot x^1(x^1)$, but this property is *first*- rather than zeroth-order, so that it falls outside the range of the variable ‘$x^1$’. (Analogously, there exists a property $\lambda x^2\,x^2(x^2)$ that is second- rather than first-order; and so on.) This in turn means that the property has no instantiation by itself, and in this way Russell’s paradox is blocked.

27 It might be replied that *being green* is in fact not intrinsic at all, but is rather the *ex*trinsic property of being disposed to act in such-and-such ways on incoming light. However, the example can be easily adjusted: simply replace ‘green’ throughout (including in the definitions of ‘grue’ and ‘bleen’) with some adjective that does designate an intrinsic property.

28 Potential objection: In Karmo’s example, God ‘necessarily knows everything’ and so necessarily exists. In that sense, one might say that God’s existence is in Karmo’s example ‘required’ even by something as seemingly unrelated as the existence of *being green*. But there is also, I think, room for a stricter sense of ‘requires’ under which that is not so.

29 See, e.g., Russell (1927: 244ff.), Quine (1960: 171), and especially Nolan (2012: 286ff.). Critics of the view include Hacker (1982) and Simons (2000).

30 See, e.g., Fitch (1971), Chisholm (1976: 128), and Tegtmeier (2000). Some philosophers, such as Bennett (1988), think of events as ‘tropes’.

31 Santayana puts this more poetically: If Heraclitus and modern physics are right in telling us that the most stable of the Pyramids is but a mass of events, this truth about substance does not dissolve substance into events that happen nowhere and to nothing [...]. If an event is to have individual identity and a place amongst other events, it must be a change which substance undergoes in one of its parts. (1923: 232) For the notion of ‘metaphysical analysis’ employed in the text, see, e.g., Dorr (2005: §13).

32 I owe this point, as well as the example of *loving the color red*, to Dan Marshall (p. c.). Against this particular example, it might be objected that *loving the color red* ought not even to be counted as a property of standing in a certain relation (such as that of loving) to anything that might properly be referred to as ‘the color red’. (Just consider any more or less successful nominalistic paraphrase of the predicate ‘loves the color red’. Thanks here to Akiko Frischhut.) However, the claim made in the text can also be supported by other examples, such as *being identical with the color red* or *having the color red as a member*, for which no nominalist paraphrases suggest themselves. As we will see below, the first of these examples has been employed by Carmichael (2016: 311) in support of exactly the same conclusion.

33 I take it that in this definition $F_1$ need not be a property but can be any sort of entity whatsoever.

34 Carmichael (p. c.) has confirmed that the behavior of his definition, as described in the previous paragraph, is not intended, and has agreed that the present suggestion seems ‘like the natural fix’.

35 Here it may be worth mentioning that the same point cannot be made, or at least not as felicitously, by using as an example the property of *having **P* *and being such that Joe Biden is self-identical* (in symbols: $\lambda x\,(P(x)\wedge I(\text {Biden},\text {Biden}))$). (Thanks to an anonymous referee for pressing me on this.) Some may be tempted to say about this property something analogous to what, in the text, has been said about *having **P* *and being such that* *P* *is self-identical*: namely that, for any *x*, to have *P* while being such that Biden is self-identical requires nothing more or less of *x* than what is required of it in order for it to have *P*. And in this way one might try to motivate an identification of *P* with $\lambda x\,(P(x)\wedge I(\text {Biden},\text {Biden}))$. However, in the first place and at least intuitively, to have *P* while being such that Biden is self-identical requires (in the general case) something more of an entity than merely to have *P*: for it requires that there be such a thing as Joe Biden. This renders the proposed identification at least *prima facie* implausible. Moreover, if the identification *were* correct, it would quickly follow that the respective property *P* involves Biden and thereby fails to be purely qualitative (assuming that Biden is either a particular himself or an intensional entity that involves a particular). By generalizing over *P*, one would thus arrive at the unwelcome result that no property at all is purely qualitative. (These points are closely related to the discussion in Sect. 2.3 above.)

36 In the analysis of $\lambda x\,(P(x)\wedge I(P,P))$ (as given by this $\lambda$-expression), *P* appears twice as an object operand in the application of the predication operation (with the identity relation playing the part of the subject operand). So *P* occurs non-predicatively in $\lambda x\,(P(x)\wedge I(P,P))$. But under the conception in question, the latter property *is* *P*. So *P* occurs non-predicatively in itself. Incidentally, it can similarly be argued that not just *P* itself but also (e.g.) the ‘negation’ of *P*, i.e., $\lambda x\,\lnot P(x)$, occurs non-predicatively in *P*. For, in the same way in which it has been argued in the text that *P* is identical with $\lambda x\,(P(x)\wedge I(P,P))$, it may be argued that *P* is identical with $\lambda x\,(P(x)\wedge I(\lambda x\,\lnot P(x),\lambda x\,\lnot P(x)))$.

37 See, e.g., Sellars (1962), Bergmann (1967; 1981), Fine (1989; 2001), O’Leary-Hawthorne and Cortens (1995), McDaniel (2009), Williamson (2013), Dorr (2016), and Russell (2018). Sider (2009; 2011) and Bacon (2019b; 2020) speak in an apparently related sense—though offering very different definitions—of ‘fundamental languages’.

38 In fact the ‘perspicuously’ may well be superfluous—in particular if, for any term *t*, variable-assignment *g*, and entity *x*, the following holds: if *t* denotes$_g$ *x* while containing a free occurrence of a term that denotes$_g$ a particular, then there exist at least one term $t'$ and variable-assignment $g'$ such that $t'$ *perspicuously* denotes$_{g'}$ *x* while containing a free occurrence of a term that denotes$_{g'}$ a particular. However, if, contrary to the recommendations of Sect. 2.3 above, one’s conception of attributes is coarse-grained enough that any property *P* is identical with $\lambda x\,(P(x)\wedge (a=a))$ for any entity *a*, then it may be reasonable to adopt a concept of perspicuous denotation under which ‘$\lambda x\,(P(x)\wedge (a=a))$’ does *not* perspicuously denote whatever it denotes. Far from being superfluous, the ‘perspicuously’ in the analysis offered in the text may then be the only thing that keeps that analysis from classifying every property as impure.

39 Cf. Quine (1976: 113); or see the relevant passage in the Introduction.

40 The Quinean notion of weak discriminability (or ‘weak discernibility’) has in recent decades gained a certain popularity in the philosophy of modern physics, where this concept has been employed in the service of investigating questions about the ‘individuality’ of subatomic particles. For an overview and critical discussion of this research program, see Bigaj (2015). Also cf. Sider (2020: §3.13). For recent discussion of different notions of discriminability from a model-theoretic perspective, see Button & Walsh(2018: §15.1).

41 Thanks to Andrew Bacon for alerting me to this fact.

42 For example, if *x* is an electron, one might let *Q* be $\lambda x,y\,(E(x)\wedge E(y))$, where *E* is the property of *being an electron*. If *x* is *not* an electron, one might let *Q* instead be $\lambda x,y\,(\lnot E(x)\wedge \lnot E(y))$.

43 Other notions of disjunctiveness have been discussed by, e.g., Sanford (1970), Langton and Lewis (1998), Clapp (2001), and Audi (2013). (For related discussion, see Plate (MSb: §3.)

44 Here I am presupposing that the ‘perspicuity’ of a term relative to a variable-assignment *g* (i.e., the question of whether the term *perspicuously* denotes$_g$ whatever it denotes$_g$) is not adversely affected by its containing the connectives ‘$\wedge$’ and ‘$\lnot$’ instead of, say, ‘$\vee$’ and the Sheffer stroke. For example, if a given property is perspicuously denoted$_g$ by ‘$\lambda x\,(P(x)\vee Q(x))$’, then, on this presupposition, it will also be perspicuously denoted$_g$ by ‘$\lambda x\,\lnot (\lnot P(x)\wedge \lnot Q(x))$’.

45 For related discussion, see Shapiro (2008: §3). Of course, on the view that *i* and $-i$ are nothing other than, respectively, the ordered pairs (0, 1) and $(0, -1)$, they *are* strongly discriminable (presumably both p. q. and s. q.): for on this view we can say that the first coordinate of *i* is less than its second coordinate, and that this is not true of $-i$. (Similarly, if *i* and $-i$ are purely qualitative intensional entities, then the properties of *being i* and *being* $-i$ may also be expected to be purely qualitative—in which case *i* and $-i$ will, again, be strongly p. q. discriminable.)
